# Training and Quality Assurance Procedures in the Asian Cohort for Alzheimer's Disease (ACAD) Study

**DOI:** 10.1002/alz70857_107295

**Published:** 2025-12-25

**Authors:** Dolly Reyes‐Dumeyer, Guerry M. Peavy, Namkhue Vo, Christina Le, Yuan Xie, Fangcong Chen, Guan Xue Chen, Anna Yao, Tiffany W Chow, Victor W Henderson, Pei‐Chuan Ho, Wai Haung Yu, Zoe D. McManus, Yian Gu, Van Ta Park, Marian Tzuang, Gyungah R Jun, Helena C Chui, Haeok Lee, Li‐San Wang, Boon Lead Tee, The Asian Cohort for Alzheimer's Disease Study (ACAD)

**Affiliations:** ^1^ G. H. Sergievsky Center, Vagelos College of Physicians and Surgeons, Columbia University, New York, NY, USA; ^2^ The Taub Institute for Research on Alzheimer's Disease and the Aging Brain, Vagelos College of Physicians & Surgeons, Columbia University, New York, NY, USA; ^3^ Department of Neurology, Vagelos College of Physicians and Surgeons, Columbia University, New York, NY, USA; ^4^ Department of Neurosciences, University of California San Diego, San Diego, CA, USA; ^5^ University of California San Diego, Department of Neurosciences, La Jolla, CA, USA; ^6^ University of California San Diego, La Jolla, CA, USA; ^7^ Columbia University, New York, NY, USA; ^8^ Memory and Aging Center, University of California San Francisco, San Francisco, CA, USA; ^9^ University of California San Francisco, San francisco, CA, USA; ^10^ Penn Neurodegeneration Genomics Center, Department of Pathology and Laboratory Medicine, University of Pennsylvania Perelman School of Medicine, Philadelphia, PA, USA; ^11^ Department of Neurology and Neurological Sciences, Stanford University, Stanford, CA, USA; ^12^ University of Toronto, Toronto, ON, Canada; ^13^ National Centralized Repository for Alzheimer's Disease and Related Dementias, Indianapolis, IN, USA; ^14^ Columbia University Irving Medical Center, New York, NY, USA; ^15^ The Gertrude H. Sergievsky Center, College of Physicians and Surgeons, Columbia University, New York, NY, USA; ^16^ Asian American Research Center on Health (ARCH), University of California San Francisco, San Francisco, CA, USA; ^17^ University of California San Francisco School of Nursing, San Francisco, CA, USA; ^18^ University of California, San Francisco, CA, USA; ^19^ Alzheimer's Disease Research Center, Boston University Chobanian & Avedisian School of Medicine, Boston, MA, USA; ^20^ Biomedical Genetics Section, Department of Medicine, Boston University Chobanian & Avedisian School of Medicine, Boston, MA, USA; ^21^ Department of Neurology, Keck School of Medicine, University of Southern California, Los Angeles, CA, USA; ^22^ Meyers College of Nursing, New York University, New York, NY, USA; ^23^ University of Pennsylvania Perelman School of Medicine, Philadelphia, PA, USA; ^24^ Memory and Aging Center, Department of Neurology, University of California San Francisco, San Francisco, CA, USA; ^25^ Global Brain Health Institute, University of California, San Francisco, San Francisco, CA, USA

## Abstract

**Background:**

The Asian Cohort for Alzheimer's Disease (ACAD) is a multi‐site collaboration to identify genetic and lifestyle risk factors for Alzheimer's disease (AD) in individuals of Chinese, Korean, and Vietnamese ancestry living in the US and Canada. To address training and quality assurance (TQA) challenges for this multilingual, multicultural cohort, the TQA core aimed to educate and train ACAD team members using a multidirectional feedback model to develop quality control measures.

**Methods:**

ACAD recruited Chinese, Korean, and Vietnamese individuals over the age of 60. Consensus diagnoses included normal cognition, mild cognitive impairment, or dementia. To ensure standardized administration of instruments and data quality, the TQA core developed a comprehensive training protocol, tailored specifically for data collection. An initial survey determined the specific role of each team member, including collection of biospecimen and clinical data, outreach, prescreening, data management and cognitive assessment. Study materials created in English, Mandarin, Cantonese, Vietnamese and Korean languages, included data collection packets, a neuropsychological instruction manual, and cognitive mock videos. For each role, ACAD members were required to demonstrate knowledge on training quizzes for certification. Quality assurance procedures (e.g., double scoring, timely re‐certification) verify data accuracy and identify error susceptibility. An all‐hands Case Diagnosis and Education Meeting (ACADEME) is co‐organized with the Clinical core to review unique cases and discuss challenging experiences.

**Results:**

To date, training completion rates for ACAD members are as follows: prescreening and outreach, (*n* = 17, 74%), data management (*n* = 13, 57%), and biospecimen collection (*n* = 14, 64%). Comparing pre‐ and post‐training quiz scores revealed significant improvements after prescreening (*p* = 0.006), outreach (*p* <0.001), data management (*p* <0.001), and biospecimen (*p* <0.001) trainings. Challenges for training investigators and staff facing a multilingual, multicultural cohort centered on developing materials with detailed instructions and designing methods to communicate and evaluate accuracy and consistency throughout the ACAD study.

**Conclusion:**

ACAD stands as one of the largest dementia cohort studies among Asians in North America. Ensuring standardized administration and data quality and integrity is crucial for generating meaningful, reproducible scientific outcomes. This study highlights the value of rigorous training for large cohort studies and underscores its impact on the broader scientific community.

